# Supplementary material for: Analyzing the Relationship between Participation in Sports and Harmful Alcohol Drinking in Early Adolescence: Two-Year Prospective Analysis
Source: Children (Basel). 2023 Jun 15;10(6):1065. doi: 10.3390/children10061065 (PMC10297176; doi:10.3390/children10061065)
Supplement: Supplementary file 1 [file children-10-01065-s001.zip › children-2460271-supplementary.pdf]

Alcohol Use Disorders Identification Test (AUDIT)

Please read the questions on the left, and encircle correct answer on the right. After answering the first question please read notation at the bottom of the Table

Table S1: AUDIT scale.

1. How often do you have a drink containing alcohol?	Never	Monthly or less	2-4 times a month	2-3 times a week	4 or more times a week
2. How many standard drinks containing alcohol do you have on a typical day when drinking?	1 or 2	3 or 4	5 or 6	7 to 9	10 or more
3. How often do you have six or more drinks on one occasion?	Never	Less than monthly	Monthly	Weekly	Daily or almost daily
4. During the past year, how often have you found that you were not able to stop drinking once you had started?	Never	Less than monthly	Monthly	Weekly	Daily or almost daily
5. During the past year, how often have you failed to do what was normally expected of you because of drinking?	Never	Less than monthly	Monthly	Weekly	Daily or almost daily
6. During the past year, how often have you needed a drink in the morning to get yourself going after a heavy drinking session?	Never	Less than monthly	Monthly	Weekly	Daily or almost daily
7. During the past year, how often have you had a feeling of guilt or remorse after drinking?	Never	Less than monthly	Monthly	Weekly	Daily or almost daily
8. During the past year, have you been unable to remember what happened the night before because you had been drinking?	Never	Less than monthly	Monthly	Weekly	Daily or almost daily
9. Have you or someone else been injured as a result of your drinking?	No		Yes, but not in the past year		Yes, during the past year
10. Has a relative or friend, doctor or other health worker been concerned about your drinking or suggested you cut down?	No		Yes, but not in the past year		Yes, during the past year

Note: If answered "Never" on first question, please proceed to questions 9 and 10
